# Effect of a 2+1 schedule of ten-valent versus 13-valent pneumococcal conjugate vaccine on pneumococcal carriage: Results from a randomised controlled trial in Vietnam

**DOI:** 10.1016/j.vaccine.2021.02.043

**Published:** 2021-04-15

**Authors:** Beth Temple, Monica Larissa Nation, Vo Thi Trang Dai, Jemima Beissbarth, Kathryn Bright, Eileen Margaret Dunne, Jason Hinds, Pham Thi Hoan, Jana Lai, Cattram Duong Nguyen, Belinda Daniela Ortika, Thanh V. Phan, Ho Nguyen Loc Thuy, Nguyen Trong Toan, Doan Y. Uyen, Catherine Satzke, Heidi Smith-Vaughan, Tran Ngoc Huu, Kim Mulholland

**Affiliations:** a Global and Tropical Health Division, Menzies School of Health Research, Charles Darwin University, Darwin, NT, Australia; b Department of Infectious Disease Epidemiology, London School of Hygiene & Tropical Medicine, London, UK; c Infection and Immunity, Murdoch Children’s Research Institute, Melbourne, VIC, Australia; d Department of Microbiology and Immunology, Pasteur Institute of Ho Chi Minh City, Ho Chi Minh City, Viet Nam; e Child Health Division, Menzies School of Health Research, Charles Darwin University, Darwin, NT, Australia; f Department of Paediatrics, The University of Melbourne, Melbourne, VIC, Australia; g Institute for Infection and Immunity, St George’s, University of London, London, UK; h BUGS Bioscience, London Bioscience Innovation Centre, London, UK; i Department of Disease Control and Prevention, Pasteur Institute of Ho Chi Minh City, Ho Chi Minh City, Viet Nam; j Department of Microbiology and Immunology, The University of Melbourne at the Peter Doherty Institute for Infection and Immunity, Melbourne, VIC, Australia

## Abstract

**Background:**

Pneumococcal conjugate vaccines (PCVs) generate herd protection by reducing nasopharyngeal (NP) carriage. Two PCVs, PCV10 and PCV13, have been in use for over a decade, yet there are few data comparing their impact on carriage. Here we report their effect on carriage in a 2+1 schedule, compared with each other and with unvaccinated controls.

**Methods:**

Data from four groups within a parallel, open-label randomised controlled trial in Ho Chi Minh City contribute to this article. Three groups were randomised to receive a 2+1 schedule of PCV10 (n = 250), a 2+1 schedule of PCV13 (n = 251), or two doses of PCV10 at 18 and 24 months (controls, n = 197). An additional group (n = 199) was recruited at 18 months to serve as controls from 18 to 24 months. NP swabs collected at 2, 6, 9, 12, 18, and 24 months were analysed (blinded) for pneumococcal carriage. This study aimed to determine if PCV10 and PCV13 have a differential effect on pneumococcal carriage, a secondary outcome of the trial. We also describe the serotype distribution among unvaccinated participants. Trial registration: ClinicalTrials.gov NCT01953510.

**Findings:**

Compared with unvaccinated controls, a 2+1 schedule of PCV10 reduced PCV10-type carriage by 45–62% from pre-booster through to 24 months of age, and a 2+1 schedule of PCV13 reduced PCV13-type carriage by 36–49% at 12 and 18 months of age. Compared directly with each other, there were few differences between the vaccines in their impact on carriage. Vaccine serotypes accounted for the majority of carriage in unvaccinated participants.

**Interpretation:**

Both PCV10 and PCV13 reduce the carriage of pneumococcal vaccine serotypes. The introduction of either vaccine would have the potential to generate significant herd protection in this population.

**Funding:**

National Health and Medical Research Council of Australia, Bill & Melinda Gates Foundation.

## Highlights

• 1st study to directly compare effect of 2+1 schedule of PCV10 or PCV13 on carriage.
• Includes an unvaccinated comparator group.
• Both PCVs reduce carriage of serotypes in the corresponding vaccine.
• May be some differences between the vaccines in their impact on carriage.
• Majority of carriage was vaccine-type, so both vaccines likely to be beneficial.

## 1 Introduction

*Streptococcus pneumoniae* (the pneumococcus) causes significant morbidity and mortality in children under five years of age, with pneumococcal pneumonia estimated to be responsible for over 380,000 deaths among that age group in 2017 [1]. There are 100 pneumococcal serotypes, and pneumococcal conjugate vaccines (PCVs) protect against a subset that most commonly cause invasive pneumococcal disease. In addition to providing direct protection to the vaccinee, PCVs result in powerful herd protection by reducing nasopharyngeal carriage and transmission of vaccine-type pneumococci to unvaccinated individuals [2].

Two PCV formulations are licenced for paediatric use. Ten-valent PCV (PCV10, Synflorix®, GSK) includes serotypes 1, 4, 5, 6B, 7F, 9V, 14, 18C, 19F, and 23F. 13-valent PCV (PCV13, Prevenar®, Pfizer) includes the same serotypes, as well as 3, 6A, and 19A. A third PCV, Pneumosil® (10-valent: serotypes 1, 5, 6A, 6B, 7F, 9V, 14, 19A, 19F, and 23F) received World Health Organization (WHO) pre-qualification in December 2019. Assessment of carriage is essential to fully evaluate the benefits of vaccination, as carriage is considered a prerequisite for disease and underpins herd protection [3], [4]. However, despite the availability of both PCV10 and PCV13 for over a decade, only one clinical trial has directly compared the effect of these vaccines on pneumococcal carriage [5]. In Papua New Guinea, a setting with high pneumococcal carriage, all participants received either PCV10 or PCV13 at 1, 2, and 3 months of age (with no unvaccinated control group), with carriage assessed at 4 and 9 months of age. Overall pneumococcal carriage transiently decreased in the PCV13 group compared with the PCV10 group, but no differences in vaccine-type carriage were observed. In Cyprus and Korea, PCV7-use was replaced with the simultaneous use of both PCV10 and PCV13. Observational studies in these two settings (among healthy children in Cyprus and among children hospitalised with respiratory infections in Korea) showed similar carriage rates with either vaccine, although a non-significant 63% reduction in the carriage of additional PCV13 serotypes was noted among PCV13-recipients compared with PCV10-recipients in Cyprus [6], [7]. A review of several other observational studies reports that introduction of either PCV10 or PCV13 leads to a reduction in vaccine-type carriage of a similar magnitude among vaccinees for the serotypes included in the vaccine [8].

As there are limited data to guide vaccine formulation choice, we undertook a randomised controlled trial of alternative PCV schedules that included a comparison of PCV10 and PCV13 in a 2+1 schedule (administered at 2, 4, and 9.5 months of age) in Ho Chi Minh City, Vietnam. Previously, we found both vaccines were safe and highly immunogenic [9]. Here, we aimed to determine if vaccine formulation had a differential effect on nasopharyngeal pneumococcal carriage and density in children during the first two years of life, comparing PCV10-recipients, PCV13-recipients, and unvaccinated controls. We also evaluate the most common serotypes carried by unvaccinated participants over time, to describe the serotypes circulating in the absence of vaccination.

## 2 Methods

### 2.1 Study design and participants

Vietnam is a lower-middle income country in South-East Asia with a population of over 95 million [10]. The burden of childhood pneumonia mortality is high, and PCV is not currently included in the national immunisation program [11]. We conducted an open label randomised controlled trial (‘The Vietnam Pneumococcal Project’), in districts 4 and 7 in Ho Chi Minh City, Vietnam. A detailed protocol describing the trial aims, study design, study population, and sample size has been published [12]. Infants were enrolled at two months of age and randomised to one of six vaccination schedules (Appendix Table S1), including a 2+1 PCV10 schedule at 2, 4, and 9.5 months of age (group C), a 2+1 PCV13 schedule at 2, 4, and 9.5 months of age (group E), and a control group that received two doses of PCV10 at 18 and 24 months of age (group F). Participants originally consented to be followed up to 18 months of age. Follow-up was later extended to 24 months of age, with an additional group (group G) enrolled at 18 months of age to serve as unvaccinated controls between 18 and 24 months. Group G participants received a single dose of PCV10 at 24 months of age. Here we describe the microbiological outcomes for participants who received a 2+1 schedule of PCV10 (group C), a 2+1 schedule of PCV13 (group E), and unvaccinated controls (groups F and G). Ethical approval was obtained from the Human Research Ethics Committee of the Northern Territory Department of Health and Menzies School of Health Research, Australia, and the Ministry of Health Ethics Committee, Vietnam.

### 2.2 Randomisation and masking

As described previously, the allocation sequence for groups A–F was generated using computerised block randomisation, stratified by district [12]. Allocation concealment was maintained using sealed envelopes with sequential study numbers on the outside of the envelope. Group G participants were recruited at 18 months of age from the study districts, concurrent with group A-F participants turning 18 months. The participants and study nurses were not blinded to group allocation, as the trial arms had different vaccination schedules. All laboratory staff were blinded to group allocation.

### 2.3 Study procedures and laboratory analyses

Study staff collected demographic information using data collection forms. Demographic data were double-entered into an EpiData v3.1 database, with validation checks completed before upload into a Microsoft Access database. Laboratory data were entered into a Microsoft Access (2–12 month time points) or Excel (18 and 24 month time points) database.

Nasopharyngeal swabs were collected at 2, 6, 9, 12, 18, and 24 months of age, and stored and tested consistent with WHO guidelines [13]. Samples collected at 2, 6, 9, and 12 months were cultured on Columbia Colistin Naladixic Acid Horse Blood agar, and *S. pneumoniae* identified based on colony morphology including α-haemolysis and susceptibility to optochin [14]. Serotyping was conducted on isolates using latex agglutination and Quellung reaction with a complete set of antisera [15]. At 18 and 24 months we performed a detailed assessment of the long-term effect of PCV on pneumococcal carriage and density using molecular methods. Samples were screened for pneumococci by quantitative real-time PCR (qPCR) targeting the autolysin (*lytA*) gene [16]. Samples with presumptive pneumococci were cultured on selective agar before molecular serotyping by microarray (Senti-SP version 1.5, BUGS Bioscience) [17]. Pneumococci were designated as non-typeable if no serotype was identified using phenotypic testing, or if microarray identified a non-encapsulated lineage (NT1, NT2, NT3a, NT3b, NT4a, NT4b, NT2/NT3b). Samples were excluded from all analyses if serotyping could not be conducted or a serotyping result could not be determined.

### 2.4 Carriage outcomes

Vaccine-type carriage was defined as carriage of a serotype contained in the vaccine formulation; PCV10-type carriage (1, 4, 5, 6B, 7F, 9V, 14, 18C, 19F, and 23F), or PCV13-type carriage (serotypes in PCV10, and 3, 6A, and 19A). Non-vaccine-type carriage was defined as carriage of a serotype not in the corresponding vaccine (excluding non-typeable pneumococci). Samples that contained both vaccine-type and non-vaccine-type serotypes were considered positive for both vaccine-type and non-vaccine-type carriage. Serotypes 15B and 15C were reported as 15B/C as these serotypes are known to interconvert [18], and ‘11F-like’ was reported as 11A [19]. Serotype-specific density at 18 and 24 months was derived by multiplying pneumococcal density (determined by *lytA* qPCR) with the relative abundance of the serotype (determined by microarray).

### 2.5 Statistical analyses

We determined the prevalence of overall pneumococcal, PCV10-type, PCV13-type, serotype 3/6A/19A (additional PCV13-type), non-PCV10-type, and non-PCV13-type carriage at 2, 6, 9, 12, 18, and 24 months for group C (2+1, PCV10), group E (2+1, PCV13), and controls. The control group varied by timepoint and was based on vaccination status: Group F (2–12 months), Groups F and G combined (18 months), or Group G (24 months). We also determined the overall probability of carriage, with participants defined as carriers if they had a positive swab at any time point. Carriage among PCV10-recipients (Group C), PCV13-recipients (Group E), and controls who were recruited at 2 months of age (Group F) was ascertained between 6 months of age (post-primary series) and 18 months of age (the time of first PCV dose in controls). Individual time point carriage prevalences and the overall probability of carriage in each of the vaccine groups were compared with controls, and a head-to-head comparison of PCV10 and PCV13 was also conducted. Prevalence ratios (PR) and 95% confidence intervals (CI) were calculated, and groups were compared using Fisher’s exact tests (5% level); one-sided when vaccine groups were compared with controls, and two-sided when vaccine groups were compared. Density data for pneumococcal carriers were log_10_-transformed and reported as log_10_ genome equivalents per ml (log_10_ GE per ml). As the transformed density data were not normally distributed, groups were compared using the non-parametric Mann-Whitney *U* test. Statistical analyses were conducted using Stata version 15.1 (StataCorp LLC). The trial is registered at ClinicalTrials.gov, number NCT01953510.

## 3 Role of the funding source

The funders of the study had no role in study design, data collection, data analysis, data interpretation, or writing of the report. The corresponding author had full access to all the data in the study and had final responsibility for the decision to submit for publication.

## 4 Results

**Fig. 1:**
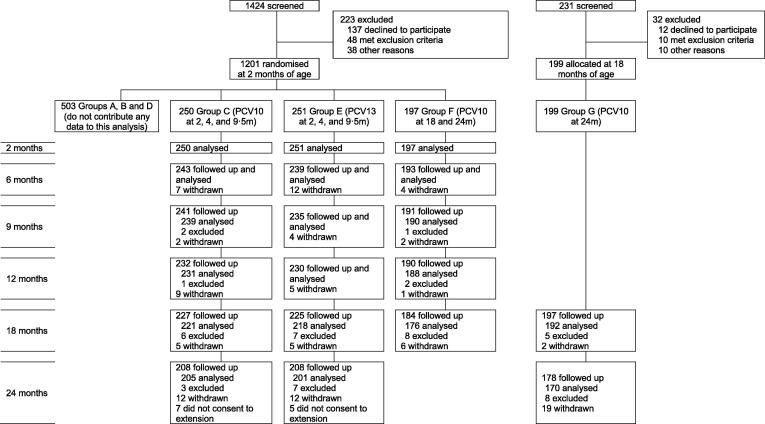
CONSORT diagram. Reasons withdrawn (n = 106): moved away and lost to follow-up (n = 67, 63%); refused a study procedure (n = 19, 18%); 16 (15%) voluntary withdrawal (n = 16, 15%); and other (n = 4, 4%). Reasons excluded: no sample (either participants missed the study visit or attended the visit but had no sample collected, n = 16); insufficient DNA for microarray (n = 24); pneumococcal carriage status could not be determined (n = 6); cultured isolate was irretrievable from freezer storage (n = 1); and excluded as a result of a protocol violation (PCV was administered outside the trial or the sample was collected after administration of PCV, n = 3). Participants who “did not consent to extension” completed the study at 18 months of age, as per the original study design. PCV = pneumococcal conjugate vaccine. PCV10 = ten-valent PCV. PCV13 = 13-valent PCV.

Between Sept 30, 2013, and Jan 9, 2015, 1201 two-month-old infants were enrolled and randomised to groups A to F (Fig. 1). Between Apr 14, 2015, and May 12, 2016, 199 18-month-old children were recruited to the additional control group (group G). Participants from groups C (2+1 PCV10, n = 250), E (2+1 PCV13, n = 251), F (controls ≤ 18 months of age, n = 197), and G (controls ≥ 18 months of age, n = 199) contribute data to this article. The groups were balanced with respect to participant demographics at baseline and to most characteristics at 18 months of age (Appendix Table S2). The exceptions were age at the 18 month visit (18.3 months in group G, compared with 18.1 months in each of groups C, E, and F, p <  0.001) and antibiotic use in the fortnight prior to the 18 month visit (20.4% in group G, compared with 12.4% in group C, 11.6% in group E, and 10.9% in group F, p = 0.020).

Of the 897 participants in this study, 106 were withdrawn and 12 did not consent to the extended follow-up beyond 18 months (Fig. 1). In all, 96.7% of participants (675/698) were followed up at 6 months, 95.6% (667/698) at 9 months, 93.4% (652/698) at 12 months, 92.9% (833/897) at 18 months, and 86.3% (594/688, excluding group F and those that did not consent to the extension) at 24 months. A total of 4103 swabs were collected, of which 4069 (99.2%) were included in the analyses. Of the 34 swabs not included, 24 had insufficient DNA for microarray, pneumococcal carriage status could not be determined in six, three were excluded due to protocol violations, and one isolate was irretrievable from freezer storage.

Overall, 616/4069 (15.1%) swabs contained capsular pneumococci. The majority of swabs (591/616, 95.9%) contained a single serotype. Of the 25 instances of multiple serotype carriage, two serotypes were identified in 23, and three serotypes were identified in each of the remaining two samples. In all, 30 different serotypes were identified (Appendix Table S3). A total of 175 non-typeable pneumococci were also identified, with two different genetic lineages detected (NT2 and NT4b, determined at 18 and 24 months only).

We examined the prevalence of pneumococcal carriage over time among PCV10-vaccinated participants, PCV13-vaccinated participants, and controls. Participant characteristics at the time of each swab were similar across groups (Table 1). The exceptions were current antibiotic use at the 9 month visit (p = 0.047), age at the 18 month visit (p = 0.016), and current symptoms of upper respiratory tract infection (URTI) at 24 months of age (p = 0.024).

**Table 1:** Characteristics of participants analysed, by time point.

	Time point	PCV10 group	PCV13 group	Control group*	p-value
**Age, months**	2 m	2.1 (1.9–2.4)	2.1 (1.9–2.4)	2.1 (2.0–2.5)	>0.999
	6 m	6.1 (5.7–6.9)	6.1 (5.7–7.0)	6.1 (5.0–6.8)	>0.999
	9 m	9.1 (9.0–10.1)	9.1 (8.8–10.1)	9.1 (9.0–11.2)	0.117
	12 m	12.1 (12.0–14.0)	12.1 (11.8–13.1)	12.1 (12.0–13.2)	>0.999
	18 m	18.1 (17.9–20.9)	18.1 (17.7–20.0)	18.2 (17.4–20.3)	0.018
	24 m	24.1 (23.9–25.9)	24.1 (23.6–28.3)	24.1 (23.4–26.9)	0.321
**Any current breastfeeding**	2 m	195/250 (78.0%)	194/250^†^ (77.6%)	140/196^†^ (71.4%)	0.208
	6 m	129/243 (53.1%)	117/239 (49.0%)	91/193 (47.2%)	0.437
	9 m	91/239 (38.1%)	88/235 (37.4%)	70/190 (36.8%)	0.966
	12 m	71/231 (30.7%)	62/230 (27.0%)	52/188 (27.7%)	0.637
	18 m	30/220^†^ (13.6%)	28/218 (12.8%)	52/368 (14.1%)	0.908
	24 m	9/205 (4.4%)	13/200^†^ (6.5%)	10/170 (5.9%)	0.636
**Presence of URTI symptoms**	2 m	18/250 (7.2%)	14/251 (5.6%)	10/197 (5.1%)	0.603
	6 m	43/243 (17.7%)	37/239 (15.5%)	27/193 (14.0%)	0.564
	9 m	38/239 (15.9%)	51/235 (21.7%)	28/190 (14.7%)	0.118
	12 m	50/231 (21.6%)	44/230 (19.1%)	34/188 (18.1%)	0.635
	18 m	23/220^†^ (10.5%)	35/218 (16.1%)	59/368 (16.0%)	0.134
	24 m	31/205 (15.1%)	44/200^†^ (22.0%)	20/170 (11.8%)	0.024
**Antibiotic use in past fortnight**	2 m	6/250 (2.4%)	12/251 (4.8%)	4/197 (2.0%)	0.178
	6 m	21/243 (8.6%)	21/239 (8.8%)	17/193 (8.8%)	0.994
	9 m	36/239 (15.1%)	41/235 (17.4%)	26/190 (13.7%)	0.551
	12 m	25/231 (10.8%)	20/230 (8.7%)	22/188 (11.7%)	0.575
	18 m	28/220^†^ (12.7%)	25/218 (11.5%)	59/368 (16.0%)	0.255
	24 m	18/205 (8.8%)	18/200^†^ (9.0%)	22/170 (12.9%)	0.337
**Current antibiotic use**	2 m	3/250 (1.2%)	6/251 (2.4%)	4/197 (2.0%)	0.602
	6 m	5/243 (2.1%)	9/239 (3.8%)	7/193 (3.6%)	0.684
	9 m	10/239 (4.2%)	18/235 (7.7%)	5/190 (2.6%)	0.047
	12 m	17/231 (7.4%)	14/230 (6.1%)	14/188 (7.4%)	0.820
	18 m	13/220^†^ (5.9%)	13/218 (6.0%)	17/368 (4.6%)	0.709
	24 m	12/205 (5.9%)	9/200^†^ (4.5%)	10/170 (5.9%)	0.788

Data are median (range) or n/N (%). p-values based on quantile regression with bootstrapped standard errors (for comparisons of medians) or chi-squared test (for comparisons of proportions). PCV = pneumococcal conjugate vaccine. PCV10 = ten-valent PCV. PCV13 = 13-valent PCV. URTI = upper respiratory tract infection (presence of runny nose and/or cough at the time of swab collection). *Data for controls comes from Group F (2–12 months), Groups F and G combined (18 months), or Group G (24 months). †Data missing for one participant.

**Fig. 2:**
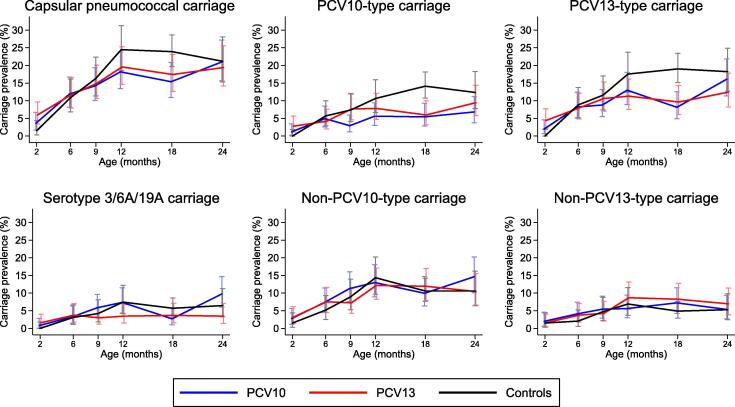
Pneumococcal carriage prevalence over time. Prevalence (95% CI) of capsular, PCV10-type, PCV13-type, serotype 3/6A/19A, non-PCV10-type, and non-PCV13-type carriage at 2, 6, 9, 12, 18, and 24 months of age, among participants who received a 2+1 schedule (at 2, 4, and 9.5 months of age) of PCV10, a 2+1 schedule of PCV13, or unvaccinated controls. CI = confidence interval. PCV = pneumococcal conjugate vaccine. PCV10 = ten-valent PCV. PCV13 = 13-valent PCV. Control group data come from: Group F (2–12 months); Groups F and G combined (18 months); or Group G (24 months).

Overall pneumococcal carriage was low at 2 months of age among all three groups, ranging from 1.5-6.0% (Fig. 2, Table 2). Carriage increased steadily to 12 months of age in all groups, peaking at 24.5% in controls and at 18.2% and 19.6% in the PCV10 and PCV13 groups, respectively. At 18 months of age, overall pneumococcal carriage was significantly lower in both vaccinated groups than controls, but this was not sustained out to 24 months of age. PCV10-type carriage was reduced by 60% among PCV10-recipients compared with controls at 9 months of age (prevalence ratio (PR) 0.40 [95% CI 0.16–0.97] p = 0.029), and this continued through to 24 months of age with reductions of 45–62% (Table 2). PCV13-type carriage was reduced by 36% and 49% among PCV13-recipients compared with controls at 12 and 18 months of age, respectively (Table 2). For both vaccines, the most profound differences were seen at 18 months of age. There was a consistent trend towards reduced carriage of the additional PCV13 serotypes (3, 6A, and 19A) among PCV13-recipients compared with controls from 12 months of age onwards, with no such trend observed among PCV10-recipients. In relation to non-vaccine-type carriage, non-PCV10-type carriage appears higher among PCV10-recipients compared with controls at 24 months of age, and non-PCV13-type carriage appears consistently higher among PCV13-recipients compared with controls from 12 months of age onwards, although these differences do not reach statistical significance.

**Table 2:** Pneumococcal carriage prevalence % (95% CI), prevalence ratio (95% CI), and Fisher’s exact p-value, by time point.

	Carriage prevalence, % (95% CI)				PCV10 vs controls			PCV13 vs controls			PCV13 vs PCV10	
	2+1 PCV10	2+1 PCV13	Controls*		Prevalence ratio (95% CI)	p-value^†^		Prevalence ratio (95% CI)	p-value^†^		Prevalence ratio (95% CI)	p-value^†^
Any pneumococcal serotype carriage												
2 m	3.6 (1.7–6.7)	6.0 (3.4–9.7)	1.5 (0.3–4.4)		2.36 (0.65–8.62)	0.146		3.92 (1.15–13.37)	0.013		1.66 (0.74–3.72)	0.296
6 m	11.9 (8.1–16.7)	11.7 (7.9–16.5)	10.9 (6.9–16.2)		1.10 (0.65–1.86)	0.426		1.08 (0.63–1.83)	0.454		0.98 (0.60–1.60)	>0.999
9 m	14.2 (10.1–19.3)	14.9 (10.6–20.1)	16.3 (11.4–22.4)		0.87 (0.56–1.36)	0.320		0.91 (0.59–1.42)	0.393		1.05 (0.68–1.62)	0.897
12 m	18.2 (13.4–23.8)	19.6 (14.6–25.3)	24.5 (18.5–31.3)		0.74 (0.51–1.08)	0.074		0.80 (0.56–1.15)	0.138		1.08 (0.74–1.57)	0.722
18 m	15.4 (10.9–20.8)	17.4 (12.6–23.1)	23.9 (19.6–28.6)		0.64 (0.45–0.92)	0.008		0.73 (0.52–1.03)	0.040		1.13 (0.74–1.73)	0.607
24 m	21.0 (15.6–27.2)	19.4 (14.2–25.6)	21.2 (15.3–28.1)		0.99 (0.67–1.47)	0.531		0.92 (0.61–1.37)	0.384		0.93 (0.63–1.36)	0.712
PCV10-type carriage												
2 m	1.2 (0.2–3.5)	2.8 (1.1–5.7)	0.0 (0.0–1.9)		(..)	0.174		(..)	0.017		2.32 (0.61–8.89)	0.339
6 m	4.9 (2.6–8.5)	4.2 (2.0–7.6)	5.7 (2.9–10.0)		0.87 (0.39–1.92)	0.443		0.73 (0.32–1.69)	0.306		0.85 (0.37–1.92)	0.828
9 m	2.9 (1.2–5.9)	7.7 (4.6–11.8)	7.4 (4.1–12.1)		0.40 (0.16–0.97)	0.029		1.04 (0.53–2.04)	0.531		2.62 (1.11–6.14)	0.024
12 m	5.6 (3.0–9.4)	7.8 (4.7–12.1)	10.6 (6.6–16.0)		0.53 (0.27–1.04)	0.044		0.74 (0.40–1.35)	0.205		1.39 (0.70–2.77)	0.360
18 m	5.4 (2.8–9.3)	6.0 (3.2–10.0)	14.1 (10.7–18.1)		0.38 (0.21–0.70)	0.001		0.42 (0.24–0.76)	0.001		1.10 (0.51–2.35)	0.840
24 m	6.8 (3.8–11.2)	9.5 (5.8–14.4)	12.4 (7.8–18.3)		0.55 (0.29–1.05)	0.049		0.77 (0.43–1.37)	0.233		1.38 (0.71–2.68)	0.368
PCV13-type carriage												
2 m	2.0 (0.7–4.6)	4.4 (2.2–7.7)	0.0 (0.0–1.9)		(..)	0.054		(..)	0.002		2.19 (0.77–6.22)	0.203
6 m	8.2 (5.1–12.4)	7.9 (4.9–12.1)	8.8 (5.2–13.7)		0.93 (0.50–1.73)	0.481		0.90 (0.48–1.69)	0.440		0.97 (0.53–1.76)	>0.999
9 m	8.8 (5.5–13.1)	10.6 (7.0–15.3)	11.6 (7.4–17.0)		0.76 (0.43–1.34)	0.213		0.92 (0.54–1.58)	0.438		1.21 (0.70–2.10)	0.537
12 m	13.0 (8.9–18.0)	11.3 (7.5–16.1)	17.6 (12.4–23.8)		0.74 (0.47–1.17)	0.123		0.64 (0.40–1.04)	0.046		0.87 (0.53–1.42)	0.669
18 m	8.1 (4.9–12.6)	9.6 (6.1–14.3)	19.0 (15.1–23.4)		0.43 (0.26–0.70)	<0.001		0.51 (0.32–0.80)	0.001		1.18 (0.65–2.16)	0.618
24 m	16.1 (11.3–21.9)	12.4 (8.2–17.8)	18.2 (12.7–24.9)		0.88 (0.57–1.38)	0.340		0.68 (0.42–1.11)	0.080		0.77 (0.48–1.25)	0.322
3/6A/19A carriage												
2 m	0.8 (0.1–2.9)	1.6 (0.4–4.0)	0.0 (0.0–1.9)		(..)	0.312		(..)	0.097		1.99 (0.37–10.78)	0.686
6 m	3.3 (1.4–6.4)	3.8 (1.7–7.0)	3.1 (1.1–6.6)		1.06 (0.37–3.00)	0.569		1.21 (0.44–3.34)	0.461		1.14 (0.45–2.91)	0.810
9 m	5.9 (3.2–9.6)	3.0 (1.2–6.0)	4.2 (1.8–8.1)		1.39 (0.60–3.25)	0.294		0.71 (0.26–1.92)	0.335		0.51 (0.21–1.24)	0.180
12 m	7.4 (4.3–11.5)	3.5 (1.5–6.7)	7.4 (4.1–12.2)		0.99 (0.50–1.95)	0.559		0.47 (0.20–1.09)	0.056		0.47 (0.21–1.07)	0.098
18 m	2.7 (1.0–5.8)	3.7 (1.6–7.1)	5.7 (3.6–8.6)		0.48 (0.20–1.16)	0.066		0.64 (0.29–1.43)	0.184		1.35 (0.48–3.83)	0.599
24 m	9.8 (6.1–14.7)	3.5 (1.4–7.0)	6.5 (3.3–11.3)		1.51 (0.74–3.06)	0.168		0.54 (0.21–1.36)	0.137		0.36 (0.15–0.83)	0.016
Non-PCV10-type carriage												
2 m	2.8 (1.1–5.7)	3.2 (1.4–6.2)	1.5 (0.3–4.4)		1.84 (0.48–7.02)	0.284		2.09 (0.56–7.79)	0.207		1.14 (0.42–3.09)	>0.999
6 m	7.4 (4.4–11.5)	7.5 (4.5–11.6)	5.2 (2.5–9.3)		1.43 (0.68–3.03)	0.229		1.45 (0.69–3.08)	0.216		1.02 (0.54–1.91)	>0.999
9 m	11.3 (7.6–16.0)	7.2 (4.3–11.3)	8.9 (5.3–13.9)		1.26 (0.71–2.25)	0.263		0.81 (0.42–1.54)	0.319		0.64 (0.36–1.14)	0.154
12 m	13.0 (8.9–18.0)	12.2 (8.2–17.1)	14.4 (9.7–20.2)		0.90 (0.56–1.47)	0.394		0.85 (0.52–1.39)	0.303		0.94 (0.58–1.52)	0.888
18 m	10.0 (6.3–14.7)	11.9 (7.9–17.0)	10.6 (7.6–14.2)		0.94 (0.57–1.54)	0.461		1.13 (0.71–1.80)	0.357		1.20 (0.70–2.05)	0.543
24 m	14.6 (10.1–20.2)	10.4 (6.6–15.5)	10.6 (6.4–16.2)		1.38 (0.80–2.39)	0.156		0.99 (0.54–1.79)	0.549		0.71 (0.42–1.20)	0.232
Non-PCV13-type carriage												
2 m	2.0 (0.7–4.6)	1.6 (0.4–4.0)	1.5 (0.3–4.4)		1.31 (0.32–5.43)	0.498		1.05 (0.24–4.62)	0.631		0.80 (0.22–2.93)	0.751
6 m	4.1 (2.0–7.4)	3.8 (1.7–7.0)	2.1 (0.6–5.2)		1.99 (0.63–6.23)	0.177		1.82 (0.57–5.81)	0.232		0.92 (0.38–2.21)	>0.999
9 m	5.4 (2.9–9.1)	4.3 (2.1–7.7)	4.7 (2.2–8.8)		1.15 (0.50–2.63)	0.460		0.90 (0.37–2.17)	0.496		0.78 (0.35–1.75)	0.670
12 m	5.6 (3.0–9.4)	8.7 (5.4–13.1)	6.9 (3.7–11.5)		0.81 (0.39–1.71)	0.365		1.26 (0.64–2.46)	0.314		1.55 (0.79–3.03)	0.212
18 m	7.2 (4.2–11.5)	8.3 (5.0–12.7)	4.9 (2.9–7.6)		1.48 (0.77–2.84)	0.158		1.69 (0.90–3.17)	0.073		1.14 (0.60–2.18)	0.724
24 m	5.4 (2.7–9.4)	7.0 (3.9–11.4)	5.3 (2.4–9.8)		1.01 (0.43–2.39)	0.581		1.32 (0.58–2.96)	0.329		1.30 (0.60–2.79)	0.541

Carriage determined by culture and latex agglutination/Quellung testing (2–12 months) and by DNA microarray (18–24 months). Samples that could not be serotyped are excluded. PCV = pneumococcal conjugate vaccine. PCV10 = ten-valent PCV. PCV13 = 13-valent PCV. * Control data sourced from Group F (2–12 month time points), Group F and G combined (18 months), or Group G (24 months). †Two-sided p-values were calculated for PCV10 vs PCV13 comparisons; one-sided p-values were calculated for comparisons with controls.

The head-to-head comparison of PCV10 and PCV13 showed few differences between vaccines (Fig. 2, Table 2). PCV10-type carriage was consistently lower among PCV10-recipients than PCV13-recipients from 9 months of age onwards (statistically significant at 9 months of age). Among PCV13-recipients, serotype 3/6A/19A carriage remained relatively constant over time, ranging from 3.0 to 3.8% between 6 and 24 months of age, and was generally lower than among PCV10-recipients from 9 months of age onwards (statistically significant at 24 months of age). Serotype 6A carriage ranged from 1.7 to 2.9%, serotype 19A carriage from 0.8 to 1.0%, and there was only one occurrence of serotype 3 carriage (at 18 months of age; Appendix Table S3). Serotype 3/6A/19A carriage fluctuated more among PCV10-recipients, ranging from 2.7 to 9.8% between 6 and 24 months of age. Serotype 6A carriage ranged from 1.6 to 6.8%, serotype 19A carriage from 0.9 to 3.0%, and there were three occurrences of serotype 3 carriage (all at 12 months of age; Appendix Table S3).

In response to lower-than-anticipated pneumococcal carriage rates we performed an additional analysis of the overall probability of carriage at any time between 6 and 18 months of age (Appendix Table S4). The overall probabilities of carriage generally reflect the trends observed over time. PCV10- and PCV13-recipients were 34% (95% CI 0–56%) and 29% (−6 to 3%) less likely to be positive for PCV10-type carriage at any time between 6 and 18 months than controls, respectively, and were 24% (−2 to 44%) and 27% (1–46%) less likely to be positive for PCV13-type carriage (Appendix Table S4). There were no differences in the overall probabilities of carriage comparing PCV10 and PCV13-recipients.

Pneumococcal density was evaluated at 18 and 24 months of age in pneumococcal carriers. Overall pneumococcal density was similar at 18 and 24 months of age, with no differences between PCV10-recipients, PCV13-recipients, and controls (Appendix Figure S1). Similarly, no differences were observed in PCV10-type, PCV13-type, serotype 3/6A/19A, non-PCV10-type, or non-PCV13-type carriage density in PCV10-recipients compared with PCV13-recipients, or between either PCV group compared with the control group.

**Fig. 3:**
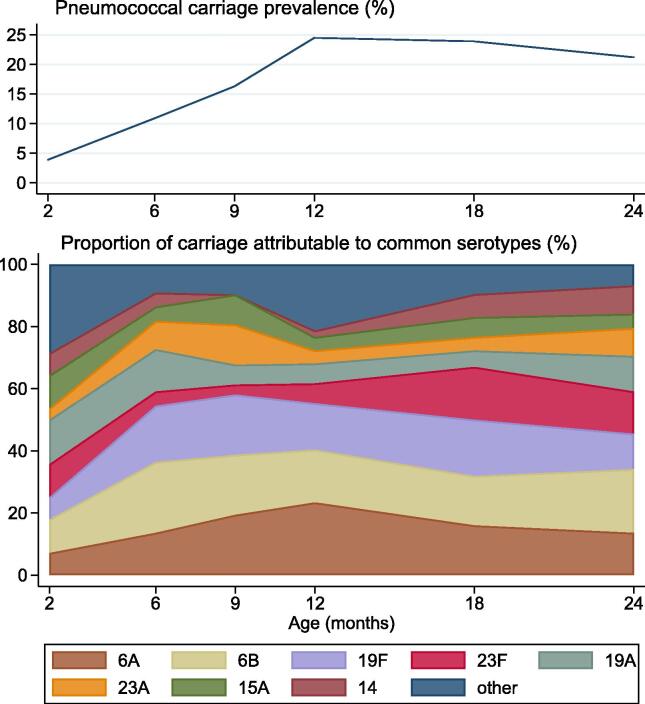
Capsular pneumococcal carriage in unvaccinated participants. The top panel shows the capsular pneumococcal carriage prevalence over time among unvaccinated participants. The bottom panel shows the proportion of carriage at each time point attributable to each of the eight most commonly carried serotypes. Data come from: all groups (2 months); Group F (2–12 months); Groups F and G combined (18 months); or Group G (24 months).

We also examined the most common serotypes carried by unvaccinated participants over time. Between 2 and 24 months of age, a total of 22 capsular serotypes were identified among unvaccinated participants, with the greatest diversity (15 different serotypes) seen at 12 months of age. Over time, the most commonly carried serotypes were 6A, 6B, 19F, 23F, 19A, 23A, 15A, and 14 (Fig. 3). These serotypes were responsible for 231 of the 266 (86.8%) pneumococci identified among unvaccinated participants. Across all time points, PCV10 serotypes accounted for 50.8% of pneumococci, and PCV13 serotypes for 75.6%.

## 5 Discussion

PCV is included universally in the national immunisation schedules of 136 countries [20]. PCV13 is used in three times as many countries as PCV10, although the total number of recipients is similar. In this paper we report the first head-to-head comparison of the effect of PCV10 and PCV13 on pneumococcal carriage in a 2+1 schedule. This schedule is becoming increasingly adopted by countries, as the booster dose may increase the duration of protection and lead to greater herd effects [21]. We show that, compared with unvaccinated controls, both vaccines reduced carriage of pneumococcal serotypes included in the corresponding vaccine. In the head-to-head comparison, PCV10 and PCV13 generally had a similar impact on carriage.

At 9 months of age (prior to the booster dose), vaccination with PCV10 resulted in a 60% reduction in PCV10-type carriage compared with unvaccinated controls. This was sustained out to 24 months of age, with reductions of 45–62%. Vaccination with PCV13 resulted in 32–49% reductions in PCV13-type carriage after the booster dose (from 12 to 24 months of age). Interestingly, vaccination with PCV10 led to consistently lower levels of PCV10-type carriage than vaccination with PCV13 from 9 months onwards, although the differences were only statistically significant at 9 months of age.

Considering the serotypes unique to PCV13, vaccination with PCV13 resulted in a consistent (albeit not statistically significant) 36–55% reduction in 3/6A/19A carriage compared with controls. Such a trend was not observed with PCV10 vaccination, despite our findings of modest immunogenicity to serotypes 6A and 19A [9], two serotypes that are known to cross-react with serotypes 6B and 19F. In the head-to-head comparison of PCV10 and PCV13, carriage of 3/6A/19A was 64% lower in the PCV13 group than the PCV10 group at 24 months of age, driven primarily by serotype 6A. This is similar to the non-significant 63% reduction in 3/6A/19A observed among PCV13-recipients compared with PCV10-recipients in Cyprus [6]. The long-term impact of a PCV10 schedule in Vietnam is unknown. Post-vaccine introduction data from elsewhere on the impact of PCV10 on carriage of serotypes 6A and 19A vary. In Brazil, carriage of both 6A and 19A had not changed three-years post-introduction of PCV10 [26], but by seven-years 6A carriage had reduced and 19A carriage had increased [27]. In Kenya, no effects were found on carriage of 6A or 19A two-years post-introduction of PCV10 [22]. Six-years post-introduction in Kenya there was still no effect on 6A carriage but 19A carriage had increased [23], similar to findings 1½-2 years post-introduction in Mozambique and three-years post-introduction in Fiji [17], [24] By contrast in Palestine, at three-years post-introduction of PCV10 there was a decrease in 3/6A/19A carriage driven by 6A, with no change in 19A [25]. It may be that the impact of PCV10 on serotypes 6A and 19A depends on the local pneumococcal epidemiology. For serotype 3, for which PCV13 is generally not considered to be effective, we observed only five instances of carriage during the trial; it is therefore not possible to determine whether PCV13 impacted serotype 3 carriage.

Serotype 19F is the most common cause of vaccine failure in children [28], and has persisted in carriage in the wake of widespread and long-term vaccination [29], [30]. Our earlier immunogenicity analyses showed that PCV10 produced better antibody and functional antibody responses to serotype 19F than PCV13 at all time points [9]. Here we found no evidence of a differential impact on 19F carriage between the two vaccines, and both PCV10 and PCV13 appear to impact the carriage of 19F to a similar degree as other vaccine types. However, the low serotype-specific carriage rates (0.9% and 2.2% at 12 months of age, 1.8% and 1.8% at 18 months of age, and 1.5% and 2.0% at 24 months of age for 19F in the PCV10 and PCV13 groups, respectively) and relatively short follow-up time make it difficult to predict the long-term consequences of our findings.

In many settings, PCV introduction has resulted in serotype replacement, whereby the reduction in vaccine-type carriage has been offset by an increase in non-vaccine-type carriage. In Vietnam, where there is still no routine PCV use, we show no definitive evidence of serotype replacement up to 24 months of age, although the increase in non-PCV10-type carriage at 24 months of age among PCV10 recipients and the trend towards higher non-PCV13-type carriage among PCV13-recipients than controls from 12 months of age onwards suggest that post-introduction surveillance for serotype replacement will be important. We did not observe any differences in pneumococcal density between groups at 18 or 24 months of age.

Data from unvaccinated participants provide information on the serotypes circulating in the absence of pneumococcal vaccination. The majority (87%) of carriage was attributable to only eight serotypes: vaccine-types 6B, 14, 19F, and 23F, additional PCV13-types 6A and 19A, and non-vaccine-types 15A and 23A. PCV10 and PCV13 serotypes represent half and three-quarters of pneumococci carried. Although it is not known what level of carriage impact is required to translate into herd protection effects, our observed reductions in vaccine-type carriage combined with the high representation of vaccine serotypes among unvaccinated participants suggest that immunisation with either vaccine is likely to impact the populations of pneumococci circulating in the community.

This trial provided a rare opportunity to evaluate the impact of vaccination with either PCV10 or PCV13 using an unvaccinated comparator group. One limitation of the study design is the use of different control groups at different time points, although few differences in characteristics were observed between groups, supporting the validity of this approach. Due to funding constraints we were not able to perform DNA microarray for all time points. However, the same method was used for all groups at any given time point. Lastly, we observed much lower pneumococcal carriage rates than anticipated; some of the non-significant differences seen between groups may therefore be due to a lack of power to detect these differences.

In conclusion, we have shown that, compared with unvaccinated controls, 2+1 schedules of PCV10 and PCV13 each reduce the carriage of pneumococcal vaccine serotypes, with the greatest impact seen at 18 months of age. There was a trend towards PCV10 having a greater impact on PCV10-type carriage than PCV13, and a trend towards PCV13 reducing serotype 3/6A/19A carriage that was not seen with PCV10. The majority of pneumococci identified from unvaccinated participants were vaccine-type, so the introduction of either PCV10 or PCV13 would have the potential to generate significant herd protection in the population.

## 6 Contributors

BT and MLN did the statistical analyses, interpreted the results with input from KM, CS, and HSV, and co-wrote the first draft of the manuscript. HSV and CS oversaw the microbiology with JB, EMD, JH and BO. VTTD managed and performed laboratory testing at the Pasteur Institute laboratory, with PTH, JL, TVP, and HNLT also contributing to laboratory testing. CDN advised on the statistical analyses and BT, MLN, JB and BO verified the underlying data. KB, NTT, and DYU were involved in the design, establishment, day-to-day management, and implementation of the trial. TNH was the site principal investigator, was involved in the design and establishment of the trial, and had overall responsibility its conduct in Vietnam. KM conceived the study, provided oversight for the conduct of the trial and the data analysis, and had overall responsibility for all aspects of the trial as the principal investigator. All authors contributed to refinement of and approved the submitted manuscript.

## 7 Data sharing

The study protocol and informed consent form have been published previously and are freely available. Data will be made publicly available in accordance with the rules set out by the Bill & Melinda Gates Foundation.

## Declaration of Competing Interest

The authors declare the following financial interests/personal relationships which may be considered as potential competing interests: All authors except JB and JH received salary support from National Health and Medical Research Council of Australia (NHMRC) and/or Bill & Melinda Gates Foundation grants. KM has received grant funding for a collaborative study on PCV impact on adult pneumonia from Pfizer. PCV10 vaccine doses were donated by GlaxoSmithKline Biologicals SA. We declare no other competing interests.

## References

[1] GBD 2017 Causes of Death Collaborators. Global, regional, and national age-sex-specific mortality for 282 causes of death in 195 countries and territories, 1980-2017: a systematic analysis for the Global Burden of Disease Study 2017. Lancet 2018;392(10159): 1736–88. 10.1016/S0140-6736(18)32203-7 PMC6227606 30496103

[2] Shiri T., Datta S., Madan J. (2017). Indirect effects of childhood pneumococcal conjugate vaccination on invasive pneumococcal disease: a systematic review and meta-analysis. The Lancet Global Health.

[3] Short K.R., Reading P.C., Wang N., Diavatopoulos D.A., Wijburg O.L. (2012). Increased nasopharyngeal bacterial titers and local inflammation facilitate transmission of *Streptococcus pneumoniae*. mBio.

[4] Vu H.T., Yoshida L.M., Suzuki M. (2011). Association between nasopharyngeal load of *Streptococcus pneumoniae*, viral coinfection, and radiologically confirmed pneumonia in Vietnamese children. Pediatr Infect Dis J.

[5] Pomat W.S., van den Biggelaar A.H.J., Wana S. (2019). Safety and immunogenicity of pneumococcal conjugate vaccines in a high-risk population: a randomized controlled trial of 10-valent and 13-valent pneumococcal conjugate vaccine in Papua New Guinean Infants. Clin Infect Dis.

[6] Hadjipanayis A., Efstathiou E., Alexandrou M. (2016). Nasopharyngeal pneumococcal carriage among healthy children in cyprus post widespread simultaneous implementation of PCV10 and PCV13 vaccines. PLoS ONE.

[7] Ahn J.G., Choi S.Y., Kim D.S., Kim K.H. (2015). Changes in pneumococcal nasopharyngeal colonization among children with respiratory tract infections before and after use of the two new extended-valency pneumococcal conjugated vaccines. Infect Dis (Lond).

[8] Pneumococcal conjugate vaccine review of impact evidence (PRIME): summary of findings from systematic review, October 2017. Geneva: World Health Organization; 2017 (https://www.who.int/immunization/sage/meetings/2017/october/3_FULL_PRIME_REPORT_2017Sep26.pdf?ua=1).

[9] Temple B., Toan N.T., Dai V.T.T. (2019). Immunogenicity and reactogenicity of ten-valent versus 13-valent pneumococcal conjugate vaccines among infants in Ho Chi Minh City, Vietnam: a randomised controlled trial. Lancet Infect Dis.

[10] World Bank. Population, total - Vietnam. 2018. https://data.worldbank.org/ [accessed 30 June 2020].

[11] Nguyen T.K., Tran T.H., Roberts C.L., Graham S.M., Marais B.J. (2017). Child pneumonia - focus on the Western Pacific Region. Paediatr Respir Rev.

[12] Temple B., Toan N.T., Uyen D.Y. (2018). Evaluation of different infant vaccination schedules incorporating pneumococcal vaccination (The Vietnam Pneumococcal Project): protocol of a randomised controlled trial. BMJ Open.

[13] Satzke C., Turner P., Virolainen-Julkunen A. (2013). Standard method for detecting upper respiratory carriage of *Streptococcus pneumoniae*: updated recommendations from the World Health Organization Pneumococcal Carriage Working Group. Vaccine.

[14] Richter S.S., Heilmann K.P., Dohrn C.L., Riahi F., Beekmann S.E., Doern G.V. (2008). Accuracy of phenotypic methods for identification of *Streptococcus pneumoniae* isolates included in surveillance programs. J Clin Microbiol.

[15] Dunne E.M., Murad C., Sudigdoadi S. (2018). Carriage of *Streptococcus pneumoniae*, *Haemophilus influenzae*, *Moraxella catarrhalis*, and *Staphylococcus aureus* in Indonesian children: a cross-sectional study. PLoS ONE.

[16] Carvalho Mda G., Tondella M.L., McCaustland K. (2007). Evaluation and improvement of real-time PCR assays targeting lytA, ply, and psaA genes for detection of pneumococcal DNA. J Clin Microbiol.

[17] Dunne E.M., Satzke C., Ratu F.T. (2018). Effect of ten-valent pneumococcal conjugate vaccine introduction on pneumococcal carriage in Fiji: results from four annual cross-sectional carriage surveys. The Lancet Global health.

[18] van Selm S., van Cann L.M., Kolkman M.A., van der Zeijst B.A., van Putten J.P. (2003). Genetic basis for the structural difference between *Streptococcus pneumoniae* serotype 15B and 15C capsular polysaccharides. Infect Immun.

[19] Manna S, Ortika BD, Dunne EM, et al. A novel genetic variant of Streptococcus pneumoniae serotype 11A discovered in Fiji. Clin Microbiol Infect 2018;24(4): 428 e1–e7. 10.1016/j.cmi.2017.06.031 PMC5869949 28736074

[20] International Vaccine Access Center (IVAC), Johns Hopkins Bloomberg School of Public Health. VIEW-hub. https://view-hub.org/ [accessed 24 April 2020].

[21] Pneumococcal conjugate vaccines in infants and children under 5 years of age: WHO position paper - February 2019. Wkly Epidemiol Rec 2019;94(08):85–104.

[22] Hammitt L.L., Akech D.O., Morpeth S.C. (2014). Population effect of 10-valent pneumococcal conjugate vaccine on nasopharyngeal carriage of *Streptococcus pneumoniae* and non-typeable *Haemophilus influenzae* in Kilifi, Kenya: findings from cross-sectional carriage studies. The Lancet Global Health.

[23] Hammitt L.L., Etyang A.O., Morpeth S.C. (2019). Effect of ten-valent pneumococcal conjugate vaccine on invasive pneumococcal disease and nasopharyngeal carriage in Kenya: a longitudinal surveillance study. Lancet.

[24] Sigauque B., Moiane B., Massora S. (2018). Early declines in vaccine type pneumococcal carriage in children less than 5 years old after introduction of 10-valent pneumococcal conjugate vaccine in Mozambique. Pediatr Infect Dis J.

[25] Abu Seir R., Azmi K., Hamdan A. (2018). Comparison of early effects of pneumococcal conjugate vaccines: PCV7, PCV10 and PCV13 on *Streptococcus pneumoniae* nasopharyngeal carriage in a population based study; The Palestinian-Israeli Collaborative Research (PICR). PLoS ONE.

[26] Brandileone M.C., Zanella R.C., Almeida S.C.G. (2016). Effect of 10-valent pneumococcal conjugate vaccine on nasopharyngeal carriage of *Streptococcus pneumoniae* and *Haemophilus influenzae* among children in Sao Paulo, Brazil. Vaccine.

[27] Brandileone M.C., Zanella R.C., Almeida S.C.G. (2019). Long-term effect of 10-valent pneumococcal conjugate vaccine on nasopharyngeal carriage of *Streptococcus pneumoniae* in children in Brazil. Vaccine.

[28] Oligbu G., Hsia Y., Folgori L., Collins S., Ladhani S. (2016). Pneumococcal conjugate vaccine failure in children: a systematic review of the literature. Vaccine.

[29] Gounder P.P., Bruce M.G., Bruden D.J. (2014). Effect of the 13-valent pneumococcal conjugate vaccine on nasopharyngeal colonization by Streptococcus pneumoniae–Alaska, 2008–2012. J Infect Dis.

[30] Madhi S.A., Nzenze S.A., Nunes M.C. (2020). Residual colonization by vaccine serotypes in rural South Africa four years following initiation of pneumococcal conjugate vaccine immunization. Expert Rev Vacc.

