# Supplementary material for: Effect of a 2+1 schedule of ten-valent versus 13-valent pneumococcal conjugate vaccine on pneumococcal carriage: Results from a randomised controlled trial in Vietnam
Source: Vaccine. 2021 Apr 15;39(16):2303–10. doi: 10.1016/j.vaccine.2021.02.043 (PMC8052188; doi:10.1016/j.vaccine.2021.02.043)
Supplement: Supplementary data 1 [file mmc1.docx]

**APPENDIX**

**Table S1:** Vaccination schedules and nasopharyngeal swabs in the Vietnam Pneumococcal Project

| **Time point** |  |  | **2m** | **3m** | **4m** |  | **6m** |  | **9m*** |  | **12m** |  | **18m** |  | **24m** |
| --- | --- | --- | --- | --- | --- | --- | --- | --- | --- | --- | --- | --- | --- | --- | --- |
| **NP swabs** |  |  | X |  |  |  | X |  | X |  | X |  | X |  | X |
| **PCV doses** |  |  |  |  |  |  |  |  |  |  |  |  |  |  |  |
| **Group** | **Schedule** | **Vaccine** |  |  |  |  |  |  |  |  |  |  |  |  |  |
| **A** | 3+1 | PCV10 | X | X | X |  |  |  | X |  |  |  |  |  |  |
| **B** | 3+0 | PCV10 | X | X | X |  |  |  |  |  |  |  |  |  |  |
| **C** | 2+1 | PCV10 | X |  | X |  |  |  | X |  |  |  |  |  |  |
| **D** | Two-dose | PCV10 | X |  |  |  | X |  |  |  |  |  |  |  |  |
| **E** | 2+1 | PCV13 | X |  | X |  |  |  | X |  |  |  |  |  |  |
| **F** | Controls | PCV10 |  |  |  |  |  |  |  |  |  |  | X |  | X |
| **G** ^†^ | Controls | PCV10 |  |  |  |  |  |  |  |  |  |  |  |  | X |

PCV = pneumococcal conjugate vaccine. PCV10 = ten-valent PCV. PCV13 = 13-valent PCV. NP = nasopharyngeal. * Booster dose of PCV administered at 9 months of age in group A and at 9·5 months of age in groups C and E. † Group G recruited at 18 months of age.

**Table S2:** Comparison of participant demographics and characteristics between groups

|  | **Group C**  (PCV10 at 2, 4 & 9·5m) | **Group E**  (PCV13 at 2, 4 & 9·5m) | **Group F**  (PCV10 at 18 & 24m) | **Group G**  (PCV10 at 24m) | p-value |
| --- | --- | --- | --- | --- | --- |
| **Participant demographics, at enrolment** |  |  |  |  |  |
| N (at enrolment) | 250 | 251 | 197 | 199 |  |
| Age (months) [median (range)] | 2·1 (1·9-2·4) | 2·1 (1·9-2·4) | 2·1 (1·9-2·5) | 18·3 (17·4-20·3) | NA |
| Sex |  |  |  |  | 0·528 |
| Male | 135 (54·0%) | 127 (50·6%) | 100 (50·8%) | 113 (56·8%) |  |
| Female | 115 (46·0%) | 124 (49·4%) | 97 (49·2%) | 86 (43·2%) |  |
| District |  |  |  |  | 0·140 |
| 4 | 112 (44·8%) | 111 (44·2%) | 87 (44·2%) | 107 (53·8%) |  |
| 7 | 138 (55·2%) | 140 (55·8%) | 110 (55·8%) | 92 (46·2%) |  |
| Birthweight (g)* [mean (sd)] | 3228 (370) | 3199 (357) | 3208 (395) | 3264 (423) | 0·326 |
| Place of delivery* |  |  |  |  | 0·424 |
| Hospital | 196 (78·7%) | 198 (79·2%) | 144 (73·1%) | 152 (76·8%) |  |
| Other | 53 (21·3%) | 52 (20·8%) | 53 (26·9%) | 46 (23·2%) |  |
| Type of delivery* |  |  |  |  | 0·063 |
| Vaginal | 160 (65·0%) | 151 (60·2%) | 121 (61·7%) | 104 (53·1%) |  |
| Elective caesarean | 43 (17·5%) | 57 (22·7%) | 34 (17·3%) | 44 (22·4%) |  |
| Emergency caesarean | 40 (16·3%) | 42 (16·7%) | 41 (20·9%) | 43 (21·9%) |  |
| Other/unknown | 3 (1·2%) | 1 (0·4%) | 0 (0·0%) | 5 (2·6%) |  |
| Cigarette smoker in house* |  |  |  |  | 0·840 |
| No | 81 (32·5%) | 86 (34·3%) | 72 (36·5%) | 70 (35·2%) |  |
| Yes | 168 (67·5%) | 165 (65·7%) | 125 (63·5%) | 129 (64·8%) |  |
| **Participant characteristics, at 18 months** |  |  |  |  |  |
| N (followed up at 18m) | 227^†^ | 225^†^ | 185^†^ | 197 |  |
| Age (months) [median (range)] | 18·1 (17·9-22·8) | 18·1 (17·7-20·8) | 18·1 (17·9-19·9) | 18·3 (17·4-20·3) | <0·001 |
| Any current breastfeeding | 31 (13·7%) | 29 (12·9%) | 21 (11·4%) | 32 (16.2%) | 0·571 |
| Presence of URTI symptoms | 24 (10·6%) | 36 (16·1%) | 28 (15·2%) | 31 (15·7%) | 0·318 |
| Antibiotic use in past fortnight | 28 (12·4%) | 26 (11·6%) | 20 (10·9%) | 40 (20·3%) | 0·023 |
| Current antibiotic use | 13 (5·8%) | 14 (6·3%) | 10 (5·4%) | 8 (4·1%) | 0·787 |

Data are n (%) unless specified. p-values based on chi-squared test (for comparisons of proportions), ANOVA (for comparisons of means), or quantile regression with bootstrapped standard errors (for comparisons of medians). PCV = pneumococcal conjugate vaccine. PCV10 = ten-valent PCV. PCV13 = 13-valent PCV. NA = not applicable, as participants intentionally recruited at different ages. URTI = upper respiratory tract infection (presence of runny nose and/or cough). * Birthweight data missing for 9 participants (3, 2, 1, and 3 from Groups C, E, F, and G, respectively); Place of delivery data missing for 3 participants (1 each from Groups C, E, and G); Type of delivery data missing for 8 participants (4, 1, and 3 from Groups C, F, and G, respectively); Cigarette smoker data missing for 1 participant from Group C. ^†^ No information other than age available at 18 months for 1 participant from Group C, and no information available at 18 months for 1 participant from each of Groups E and F.

**Table S3:** Serotype-specific carriage prevalence, by time point

| **Vaccine serotypes** | **Group** | **Carriage prevalence (n/N)** | | | | | |
| --- | --- | --- | --- | --- | --- | --- | --- |
|  |  | **2 months** | **6 months** | **9 months** | **12 months** | **18 months** | **24 months** |
| **PCV10-types** |  |  |  |  |  |  |  |
| **6B** | PCV10 | 0·4 (1/250) | 1·2 (3/243) | 1·3 (3/239) | 2·6 (6/231) | 0·9 (2/221) | 1·5 (3/205) |
|  | PCV13 | 0·8 (2/251) | 2·5 (6/239) | 3·8 (9/235) | 3·0 (7/230) | 1·4 (3/218) | 3·5 (7/201) |
|  | Control* |  | 2·6 (5/193) | 3·2 (6/190) | 4·3 (8/188) | 4·1 (15/368) | 5·3 (9/170) |
| **14** | PCV10 | 0·4 (1/250) |  |  |  |  |  |
|  | PCV13 | 0·4 (1/251) |  |  | 0·4 (1/230) | 0·5 (1/218) | 0·5 (1/201) |
|  | Control* |  | 0·5 (1/193) |  | 0·5 (1/188) | 1·9 (7/368) | 2·4 (4/170) |
| **19F** | PCV10 |  | 0·4 (1/243) | 0·4 (1/239) | 0·9 (2/231) | 1·8 (4/221) | 1·5 (3/205) |
|  | PCV13 | 0·8 (2/251) | 1·3 (3/239) | 2·1 (5/235) | 2·2 (5/230) | 1·8 (4/218) | 2·0 (4/201) |
|  | Control* |  | 2·1 (4/193) | 3·2 (6/190) | 3·7 (7/188) | 4·6 (17/368) | 2·9 (5/170) |
| **23F** | PCV10 | 0·4 (1/250) | 3·3 (8/243) | 1·3 (3/239) | 2·2 (5/231) | 2·7 (6/221) | 3·4 (7/205) |
|  | PCV13 | 0·8 (2/251) | 0·4 (1/239) | 1·7 (4/235) | 2·2 (5/230) | 2·3 (5/218) | 3·0 (6/201) |
|  | Control* |  | 0·5 (1/193) | 0·5 (1/190) | 1·6 (3/188) | 4·3 (16/368) | 3·5 (6/170) |
| **Additional PCV13-types** |  |  |  |  |  |  |  |
| **3** | PCV10 |  |  |  | 1·3 (3/231) |  |  |
|  | PCV13 |  |  |  |  | 0·5 (1/218) |  |
|  | Control* |  |  |  |  | 0·3 (1/368) |  |
| **6A** | PCV10 |  | 1·6 (4/243) | 3·3 (8/239) | 3·0 (7/231) | 1·8 (4/221) | 6·8 (14/205) |
|  | PCV13 | 0·8 (2/251) | 2·9 (7/239) | 2·1 (5/235) | 1·7 (4/230) | 1·8 (4/218) | 2·5 (5/201) |
|  | Control* |  | 1·6 (3/193) | 3·2 (6/190) | 5·9 (11/188) | 4·1 (15/368) | 3·5 (6/170) |
| **19A** | PCV10 | 0·8 (2/250) | 1·6 (4/243) | 2·9 (7/239) | 3·0 (7/231) | 0·9 (2/221) | 2·9 (6/205) |
|  | PCV13 | 0·8 (2/251) | 0·8 (2/239) | 0·9 (2/235) | 1·7 (4/230) | 1·4 (3/218) | 1·0 (2/201) |
|  | Control* |  | 1·6 (3/193) | 1·1 (2/190) | 1·6 (3/188) | 1·4 (5/368) | 2·9 (5/170) |
| **Other vaccine-types**† | PCV10 |  |  |  |  | 0·5 (1/221) | 0·5 (1/205) |
|  | PCV13 |  |  |  |  |  | 0·5 (1/201) |
|  | Control* |  | 0·5 (1/193) | 0·5 (1/190) | 0·5 (1/188) |  |  |

| **Non-vaccine serotypes** | **Group** | **Carriage prevalence (n/N)** | | | | | |
| --- | --- | --- | --- | --- | --- | --- | --- |
|  |  | **2 months** | **6 months** | **9 months** | **12 months** | **18 months** | **24 months** |
| **15A** | PCV10 | 0·8 (2/250) | 0·4 (1/243) | 1·3 (3/239) | 2·2 (5/231) | 1·8 (4/221) | 1·5 (3/205) |
|  | PCV13 |  | 0·4 (1/239) | 0·9 (2/235) | 1·3 (3/230) | 2·8 (6/218) | 2·0 (4/201) |
|  | Control* | 0·5 (1/197) | 0·5 (1/193) | 1·6 (3/190) | 1·1 (2/188) | 1·6 (6/368) | 1·2 (2/170) |
|  |  |  |  |  |  |  |  |
| **15B/C** | PCV10 |  |  | 0·4 (1/239) | 0·4 (1/231) | 2·3 (5/221) | 2·4 (5/205) |
|  | PCV13 | 0·4 (1/251) | 0·4 (1/239) |  | 2·2 (5/230) | 2·8 (6/218) | 1·0 (2/201) |
|  | Control* |  |  |  | 2·1 (4/188) | 0·5 (2/368) | 1·2 (2/170) |
|  |  |  |  |  |  |  |  |
| **23A** | PCV10 | 0·4 (1/250) | 0·8 (2/243) |  |  | 0·9 (2/221) | 0·5 (1/205) |
|  | PCV13 |  | 0·4 (1/239) | 1·3 (3/235) | 0·4 (1/230) | 0·5 (1/218) | 0·5 (1/201) |
|  | Control* |  | 1·0 (2/193) | 2·1 (4/190) | 1·1 (2/188) | 1·1 (4/368) | 2·4 (4/170) |
|  |  |  |  |  |  |  |  |
| **34** | PCV10 |  |  | 0·8 (2/239) | 1·3 (3/231) | 0·5 (1/221) | 0·5 (1/205) |
|  | PCV13 | 0·4 (1/251) | 0·8 (2/239) | 0·9 (2/235) | 1·3 (3/230) | 0·5 (1/218) | 1·5 (3/201) |
|  | Control* |  | 0·5 (1/193) | 1·1 (2/190) | 0·5 (1/188) | 1·1 (4/368) |  |
|  |  |  |  |  |  |  |  |
| **Other non-vaccine-types**‡ | PCV10 | 0·8 (2/250) | 2·9 (7/243) | 2·5 (6/239) | 2·2 (5/231) | 1·8 (4/221) | 0·5 (1/205) |
|  | PCV13 | 0·8 (2/251) | 1·7 (4/239) | 1·3 (3/235) | 3·9 (9/230) | 1·8 (4/218) | 2·0 (4/201) |
|  | Control* | 1·0 (2/197) |  |  | 2·1 (4/188) | 0·5 (2/368) | 0·6 (1/170) |
|  |  |  |  |  |  |  |  |

Blank cells indicate no carriage. PCV = pneumococcal conjugate vaccine. PCV10 = ten-valent PCV. PCV13 = 13-valent PCV. * Control data sourced from Group F (2–12 month time points), Group F and G combined (18 months), or Group G (24 months). † The **6 other vaccine-types** comprised: **4 x serotype 4** (1at 9m [Group F], 1 at 12m [Group F], 2 at 24m [1 in Group C, 1 in Group E]); **1 x serotype 18C** (at 6m [Group F]); and **1 x serotype 9V** (at 18m [Group C]). ‡ The **46 other non-vaccine-types** comprised: **25 x serotype 11A** (2 at 2m [Group C], 4 at 6m [3 in Group C, 1 in Group E], 5 at 9m [4 in Group C, 1 in Group E], 8 at 12m [4 in Group C, 3 in Group E, 1 in Group F], 4 at 18m (2 in Group C, 1 in Group E, 1 in Group F/G], and 2 at 24m [Group E]); **7 x serotype 35B** (2 at 6m [1 in Group C, 1 in Group E], 1 at 9m [Group E], 1 at 12m [Group E], 2 at 18m [1 in Group C, 1 in Group E], and 1 at 24m [Group G]); **4 x serotypes 7C, 13, and 19C** (7C: 1 at 2m [Group E], 1 at 6m [Group E], 1 at 12m [Group F], 1 at 24m [Group E]; 13: 1 at 9m [Group C], 2 at 18m [1 in Group C, 1 in Group F/G], 1 at 24m [Group E]; 19C: 1 at 2m [Group F], 3 at 12m [1 in Group C, 2 in Group E]); **3 x serotypes 6C and 17F** (6C: 1 at 2m, 9m, and 12m [all in Group E]; 17F: 2 at 12m [1 in Group E, 1 in Group F], 1 at 18m [Group E]); **2 x serotypes 16F and 35F** (16F: 1 at 9m [Group C], 1 at 12m [Group E]; 35F: 2 at 6m [1 in Group C, 1 in Group E]); and **1 x serotypes 8** (12m [Group F]), **19B** (2m [Group F]), **20** (9m [Group C]), **35A** (18m [Group E]), **37** (6m [Group C]), and **38** (24m [Group C]).

**Table S4:** Overall probability of carriage between 6 and 18 months of age

|  | Carriage prevalence, % (95% CI) | | |  | PCV10 vs Controls | |  | PCV13 vs Controls | |  | PCV13 vs PCV10 | |
| --- | --- | --- | --- | --- | --- | --- | --- | --- | --- | --- | --- | --- |
|  | 2+1 PCV10 | 2+1 PCV13 | Controls* |  | Prevalence ratio (95% CI) | p-value† |  | Prevalence ratio (95% CI) | p-value† |  | Prevalence ratio (95% CI) | p-value† |
| Any pneumococcal serotype carriage | 36·6 (30·6-43·0) | 36·4 (30·3-42·8) | 44·0 (36·9-51·3) |  | 0·83 (0·66-1·05) | 0·071 |  | 0·83 (0·66-1·04) | 0·065 |  | 0·99 (0·79-1·26) | >0·999 |
| PCV10-type carriage | 14·0 (9·9-19·0) | 15·1 (10·8-20·2) | 21·2 (15·7-27·7) |  | 0·66 (0·44-1·00) | 0·031 |  | 0·71 (0·47-1·06) | 0·062 |  | 1·08 (0·70-1·66) | 0·796 |
| PCV13-type carriage | 24·7 (19·4-30·6) | 23·8 (18·6-29·8) | 32·6 (26·1-39·7) |  | 0·76 (0·56-1·02) | 0·042 |  | 0·73 (0·54-0·99) | 0·028 |  | 0·97 (0·70-1·32) | 0·833 |
| 3/6A/19A carriage | 12·3 (8·5-17·2) | 9·6 (6·2-14·1) | 15·0 (10·3-20·9) |  | 0·82 (0·51-1·32) | 0·25 |  | 0·64 (0·38-1·07) | 0·059 |  | 0·78 (0·47-1·30) | 0·383 |
| Non-PCV10-type carriage | 24·7 (19·4-30·6) | 24·7 (19·4-30·7) | 27·5 (21·3-34·3) |  | 0·90 (0·65-1·24) | 0·292 |  | 0·90 (0·65-1·24) | 0·293 |  | 1·00 (0·73-1·37) | >0·999 |
| Non-PCV13-type carriage | 15·2 (11·0-20·4) | 16·3 (11·9-21·6) | 13·0 (8·6-18·5) |  | 1·18 (0·73-1·88) | 0·297 |  | 1·26 (0·79-2·01) | 0·2 |  | 1·07 (0·71-1·62) | 0·803 |

Overall probability of carriage defined as the percentage of participants with any positive swab between 6 and 18 months of age. PCV = pneumococcal conjugate vaccine. PCV10 = ten-valent PCV. PCV13 = 13-valent PCV. * Control data restricted to Group F. † Two–sided Fisher’s exact test used for PCV10 vs PCV13 comparisons; one–sided Fisher’s exact test used for comparisons with controls.
