# Supplementary material for: Effect of a 2+1 schedule of ten-valent versus 13-valent pneumococcal conjugate vaccine on pneumococcal carriage: Results from a randomised controlled trial in Vietnam
Source: Vaccine. 2021 Apr 15;39(16):2303–10. doi: 10.1016/j.vaccine.2021.02.043 (PMC8052188; doi:10.1016/j.vaccine.2021.02.043)
Supplement: Supplementary data 2 [file mmc2.pdf]

Appendix Figure S1: Pneumococcal carriage density among pneumococcal carriers a) at 18 months of age and b) at 24 months of age

Median (IQR) density (log_{10} genome equivalents per ml) of capsular, PCV10-type, PCV13-type, serotype 3/6A/19A, non-PCV10-type, and non-PCV13-type carriage, among pneumococcal carriers at a) 18 months of age and b) 24 months of age who received a 2+1 schedule of PCV10, a 2+1 schedule of PCV13, or unvaccinated controls. IQR = interquartile range. PCV = pneumococcal conjugate vaccine. PCV10 = ten-valent PCV. PCV13 = 13-valent PCV. IQR = inter-quartile range. Control group data come from: Group F (2-12 months); Groups F and G combined (18 months); or Group G (24 months). ● denotes a datapoint greater than the 75th percentile plus 1·5 times the IQR.

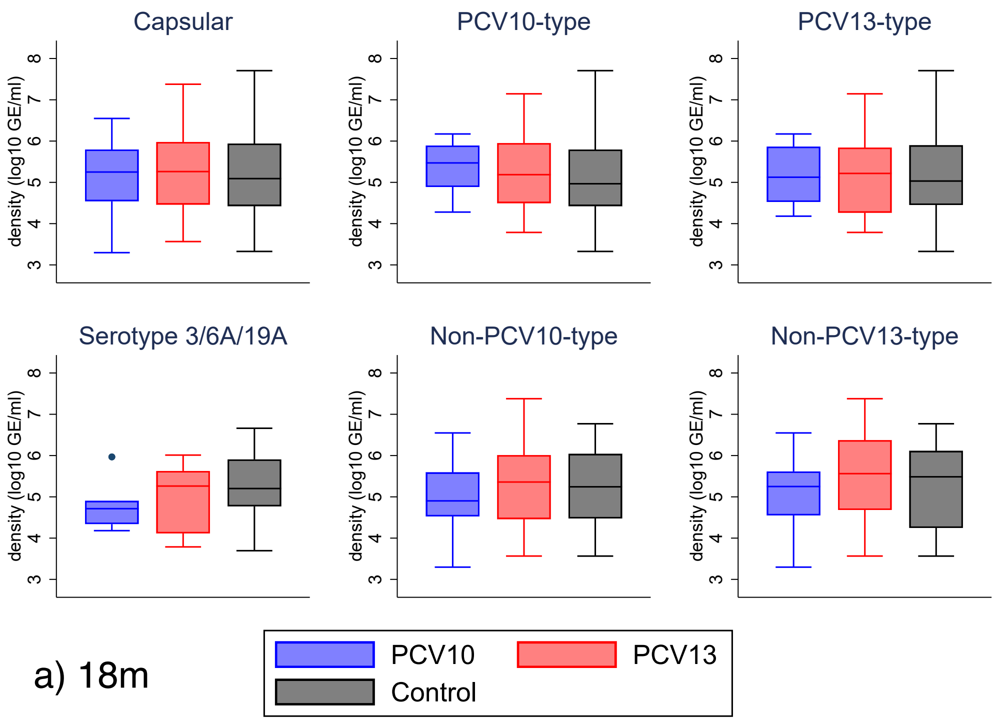
Capsular
PCV10-type
PCV13-type
Serotype 3/6A/19A
Non-PCV10-type
Non-PCV13-type
density (log10 GE/ml)
3
4
5
6
7
8
PCV10
PCV13
Control
a) 18m

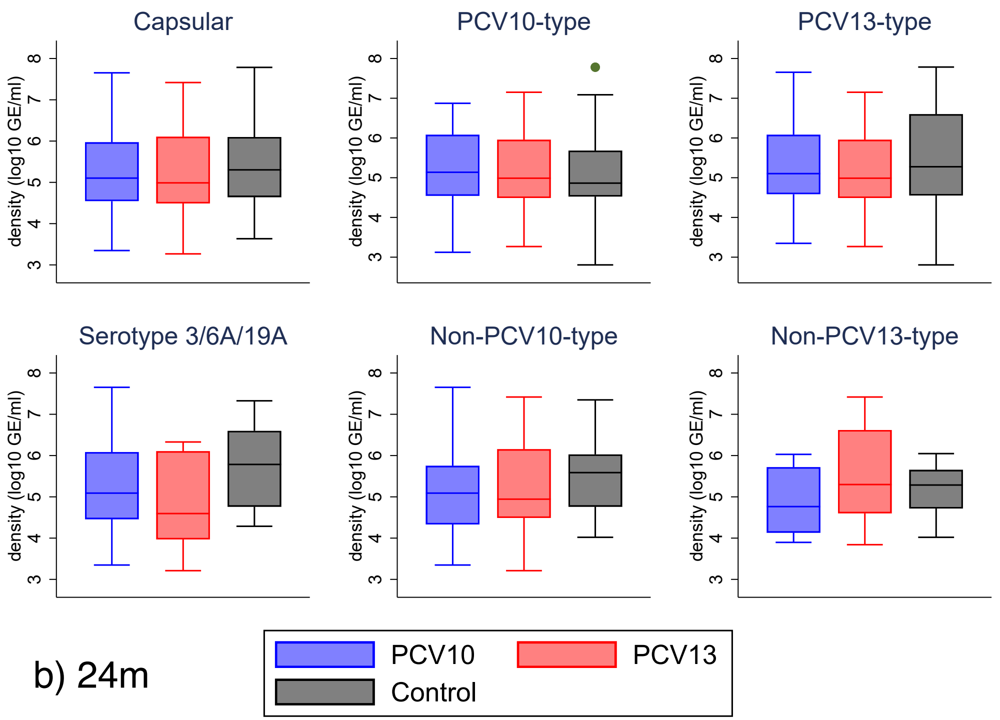
Capsular
PCV10-type
PCV13-type
Serotype 3/6A/19A
Non-PCV10-type
Non-PCV13-type
density (log10 GE/ml)
3
4
5
6
7
8
PCV10
PCV13
Control
b) 24m
